# Challenges associated with managing treatment complications in an older patient with cardiac amyloidosis

**DOI:** 10.1186/s43044-024-00507-0

**Published:** 2024-06-18

**Authors:** Soo Yeon An, Yujin Yang

**Affiliations:** 1 Division of Cardiology, Department of Internal Medicine, Chungnam National University Hospital, College of Medicine, Chungnam National University, Daejeon, Republic of Korea; 2 Department of Medical Sciences, College of Medicine, Chungnam National University, Daejeon, Republic of Korea

**Keywords:** Wild-type transthyretin amyloidosis (ATTRwt), Heart failure with preserved ejection fraction (HFpEF), Elderly, Personalized medicine, Treatment challenges

## Abstract

**Background:**

Amyloidosis, particularly wild-type transthyretin amyloidosis (ATTRwt), is an increasingly recognized cause of heart failure with preserved ejection fraction in the aging population. The complexity of managing ATTRwt in older patients underscores the necessity for individualized treatment approaches, yet clinical guidelines are lacking. This case report contributes to the understanding of ATTRwt management in the elderly, emphasizing the intricacies of medication tolerance and therapeutic decision-making.

**Case presentation:**

An 83-year-old Korean man with a history of hypertension presented with dyspnea and peripheral edema. Investigations including electrocardiography, transthoracic echocardiography, cardiac magnetic resonance, and Technetium pyrophosphate scintigraphy led to a diagnosis of ATTRwt cardiac amyloidosis. Initial management with heart failure medications, including an angiotensin-converting enzyme inhibitor, diuretic, and mineralocorticoid receptor antagonist, was modified due to evolving clinical presentations, such as hypotension and onset of atrial fibrillation. Challenges included intolerance to beta-blockers and bleeding complications from direct oral anticoagulant therapy. The patient’s treatment journey highlighted the need for personalized management strategies in older ATTRwt patients.

**Conclusions:**

This case illustrates the challenges in diagnosing and managing ATTRwt amyloidosis in the elderly, particularly the complexities in medication management due to the patient’s age, comorbid conditions, and side effects. It underscores the importance of a tailored approach in managing ATTRwt in older populations and highlights the need for ongoing research and development of treatment strategies tailored to this demographic.

## Background

Amyloidosis is a multifaceted multisystemic disorder, increasingly recognized in aging populations with heart failure and preserved ejection fraction (HFpEF) [1]. Wild-type transthyretin amyloidosis (ATTRwt) is an age-dependent form of systemic amyloidosis characterized by the deposition of amyloid fibrils comprising the transthyretin (TTR) protein in the heart [2]. Cardiac manifestations typically include left ventricular (LV) wall thickening, diastolic dysfunction, bradyarrhythmia, atrial fibrillation (AF), and the clinical presentation of HFpEF [3]. A clinical guideline for the individualized approach to managing ATTRwt in the older population is lacking. Hence, every medication should be meticulously evaluated based on the patient's presentation and response. Here, we present challenges in drug therapy for ATTRwt old patients, which clinician can commonly encounter.

## Case presentation

An 83-year-old Korean man with a previous history of hypertension treated with anti-hypertensive medication, who was referred to the Cardiology Clinic because of a six-month history of dyspnea (New York Heart Association functional class III) and peripheral edema. Upon examination, his blood pressure and heart rate were 108/70 mmHg and 75 bpm, respectively. Additionally, mild pretibial pitting edema was observed.

**Fig. 1:**
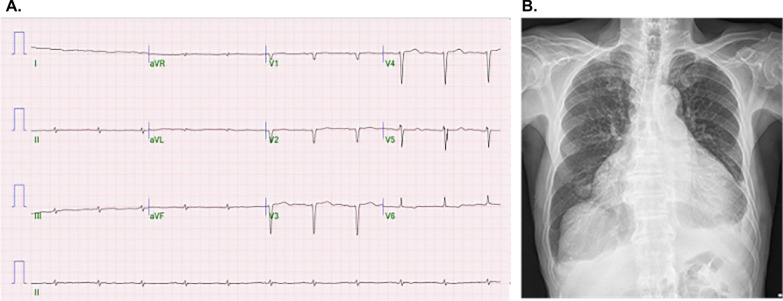
**A** Electrocardiography shows normal sinus rhythm with low QRS voltage and Q waves in V2–V4. **B** Cardiomegaly and pleural effusion are noted on the chest radiograph

**Fig. 2:**
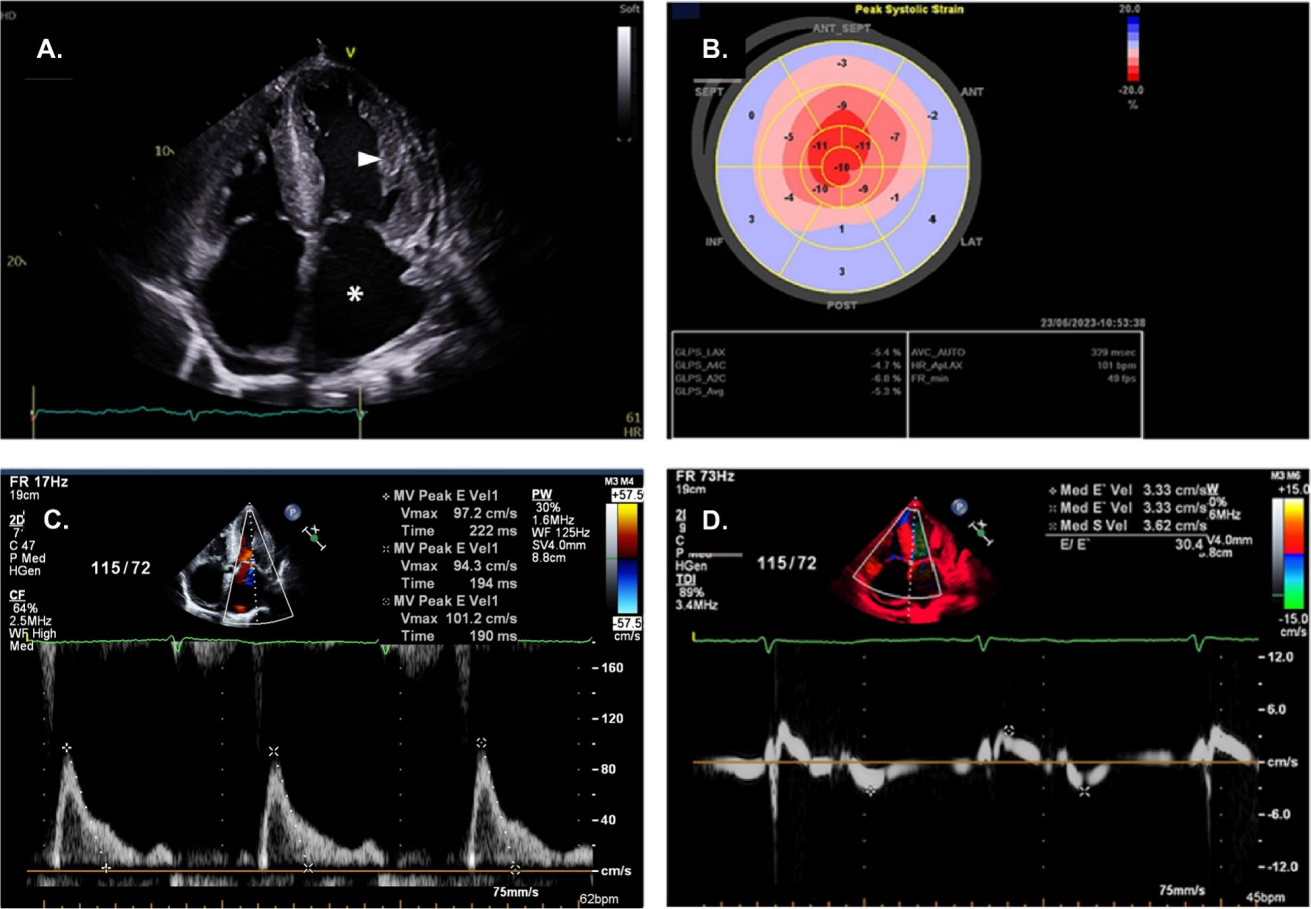
**A** Transthoracic echocardiography (TTE) reveals enlargement of the left ventricle and left atrium (white asterisk), increased LV diameter and a thickened LV wall with a granular sparkling texture of the myocardium (white arrowhead), and pericardial effusion. **B** The longitudinal strain is globally reduced on the bull’s eye plot with relative apical sparing. **C** Transmitral flow evaluation reveals a restrictive pattern with a high mitral inflow velocity of 100 cm/s and **D** an E/e′ ratio of 29

Electrocardiography revealed normal sinus rhythm with low QRS voltage and Q waves in V2–V4, indicating a pseudo-infarct pattern (Fig. 1A). Suspecting heart failure owing to long-standing hypertension with cardiomegaly and pleural effusion on chest radiography (Fig. 1B), transthoracic echocardiography (TTE) was performed. It revealed an LV end-diastolic diameter of 51 mm and a thickened LV wall of 15 mm with an LV ejection fraction of 48%. In addition, it showed a granular sparkling texture of the myocardium and minimal pericardial effusion (Fig. 2A). The longitudinal strain showed globally reduced strain values in the bull’s eye plot with relative apical sparing (Fig. 2B). Transmitral flow evaluation revealed a restrictive pattern with a high mitral inflow velocity of 100 cm/s and an E/e’ ratio of 29 (Fig. 2C, D). Given the clinical presentation with the aforementioned electrocardiographic and echocardiographic findings, cardiac amyloidosis was suspected.

To relieve symptoms and volume overload, the patient was prescribed a combination of heart failure medications, including an angiotensin-converting enzyme inhibitor (ACEI, perindopril 2.5 mg QD), diuretic agent (furosemide 20 mg QD), and mineralocorticoid receptor antagonist (MRA, spironolactone 25 mg QD). Over the one-month follow-up period, the patient’s cardiac symptoms gradually improved. Furthermore, as his blood pressure decreased to 90/60 mmHg at the one-month follow-up visit, perindopril was discontinued and switched to an angiotensin receptor blocker (ARB, valsartan 40 mg QD), expecting patient’s better tolerability with ARB.

To confirm and classify the type of cardiac amyloidosis, the patient underwent advanced imaging evaluations, including cardiac magnetic resonance (CMR) and Technetium pyrophosphate (99mTc-PYP) scintigraphy.

**Fig. 3:**
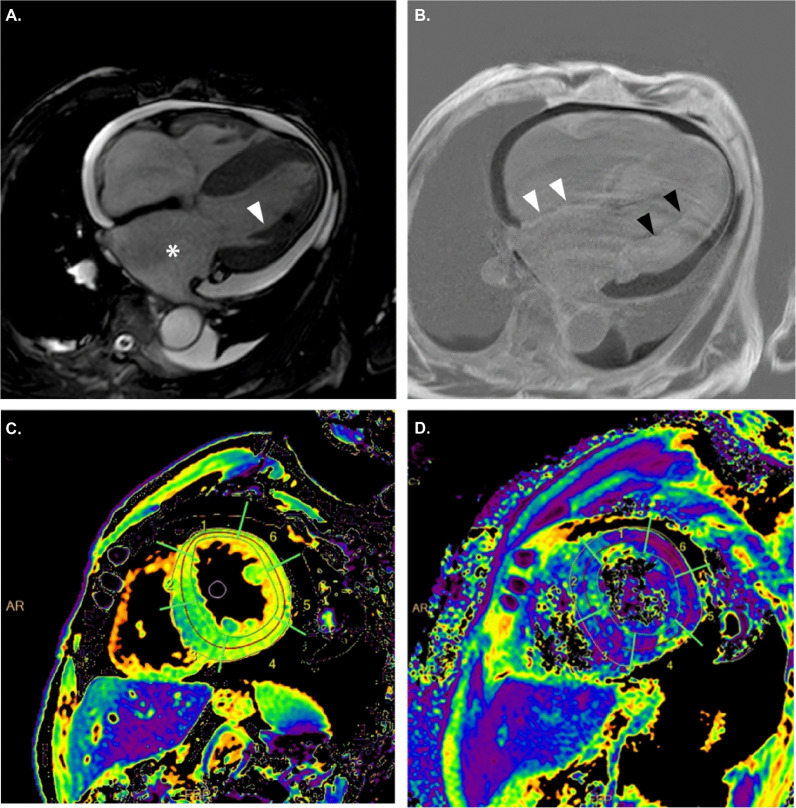
**A** Cardiac magnetic resonance (CMR) images show concentric left ventricular (LV) hypertrophy (white arrowhead) and atrial dilatation (white asterisk) on the axial plane view. **B** Late gadolinium enhancement is present in the subendocardium of the LV (black arrowheads) and atrial walls (white arrowheads) on the horizontal long-axis. **C** The lack of myocardial signal suppression at various TI times suggests amyloid deposition in the LV on T1 and **D** T2 mapping

CMR images showed concentric LV hypertrophy and atrial dilatation (Fig. 3A). Late gadolinium enhancement was detected in the subendocardium of the LV and atrial walls (Fig. 3B). The lack of myocardial signal suppression at various TI times suggested amyloid deposition in the LV on T1 (Fig. 3C) and T2 mapping (Fig. 3D). CMR findings were consistent with cardiac amyloidosis.

**Fig. 4:**
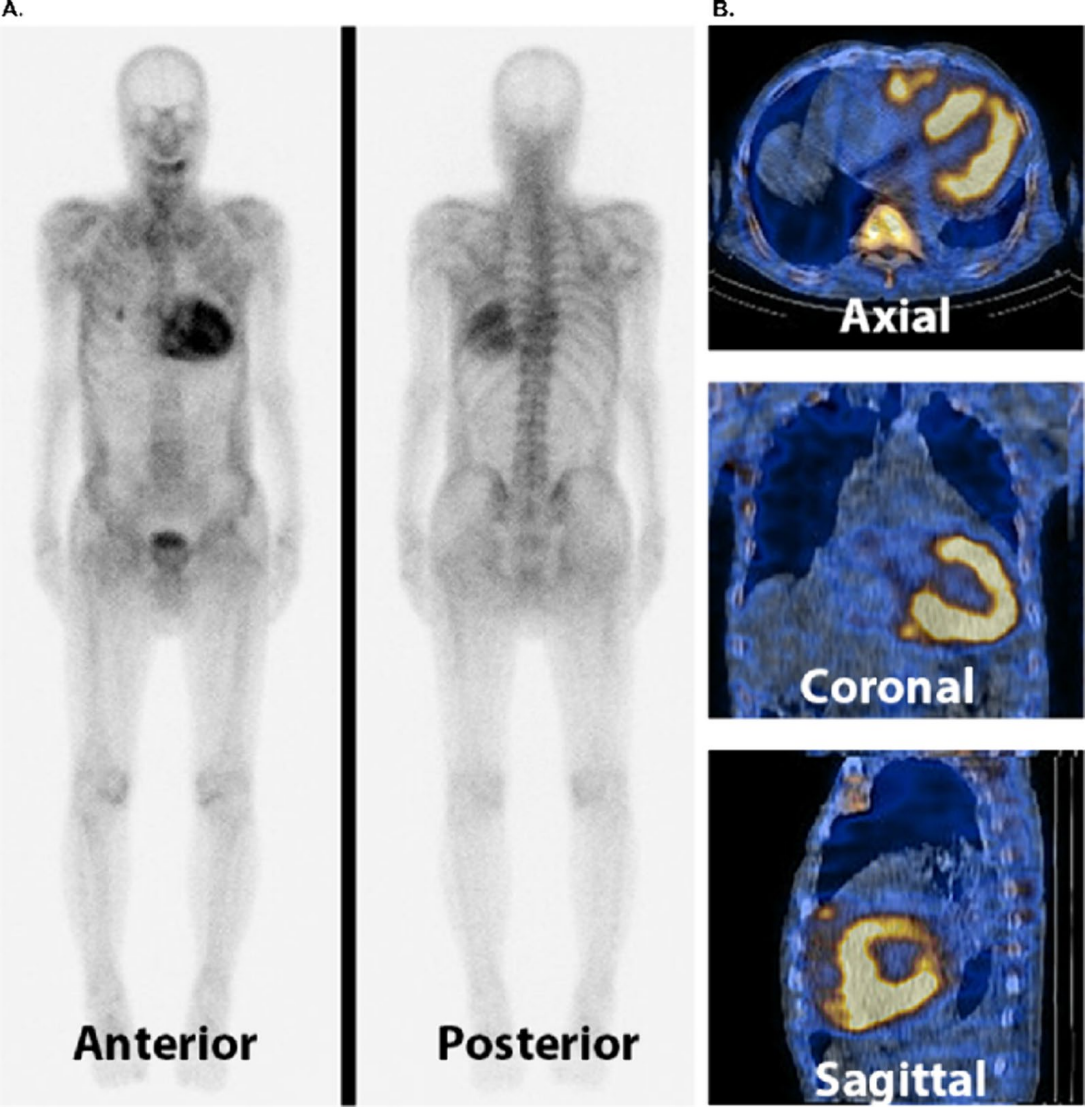
**A** Technetium pyrophosphate (99mTc-PYP) scintigraphy reveals marked myocardial uptake (Perugini grade 3) of 99mTc-PYP with low skeletal uptake on the whole-body images of the anterior and posterior sides. **B** Single-photon emission computed tomography-computed tomography images of the heart in the axial, coronal, and sagittal planes show increased myocardial uptake

99mTc-PYP scintigraphy findings supported the diagnosis of ATTR cardiac amyloidosis. Marked myocardial uptake (Perugini grade 3) of 99mTc-PYP, greater than that in bones, was found with attenuated skeletal uptake on whole-body images (Fig. 4A). Single-photon emission computed tomography images of the heart in the axial, coronal, and sagittal planes showed increased myocardial uptake (Fig. 4B).

Laboratory tests revealed no evidence of monoclonal gammopathy (Table 1). Genetic testing revealed no *TTR* gene mutations; therefore, the patient was diagnosed with ATTRwt amyloidosis. The patient exhibited no clinical signs of autonomic or peripheral neuropathy. Further evaluation, including endomyocardial biopsy, was recommended; however, the patient and his family declined and only wanted symptom control because of his advanced age. Tafamidis, a TTR stabilizer, was recommended for disease management and better prognosis. However, because of its high cost, the patient declined the treatment.

**Table 1:** Initial blood laboratory test results

Laboratory test	Test value	Reference value
White blood cell count	3.87 × 10^3^/uL	3.8–10.0 × 10^3^/uL
Hemoglobin	11.0 g/dL	13.5–17.5 g/dL
Platelet count	73 × 10^3^/uL	130–400 × 10^3^/uL
Creatinine	0.83 mg/dL	0.6–1.1 mg/dL
BUN	16.0 mg/dL	8–20 mg/dL
NT-proBNP	1710 pg/mL	< 125 pg/mL
Troponin I	120.7 pg/mL	2.3–17.5 pg/mL
B2-MG	3.78 mg/L	0–3.0 mg/L
Free T4	1.04 ng/dL	0.7–1.48 ng/dL
TSH	3.8762 uIU/mL	0.35–4.94 uIU/mL
IgG	1715 mg/dL	680–1620 mg/dL
IgA	343 mg/dL	84–438 mg/dL
IgM	96 mg/dL	57–288 mg/dL
Free Kappa light chain	52.86 mg/L	3.30–19.4 mg/L
Free Lambda light chain	30.32 mg/L	5.71–26.30 mg/L
Free K/L ratio	1.744%	0.26–1.65%
Free Kappa + Lambda	83.183%	
Free Kappa – Lambda	22.549%	

**Fig. 5:**
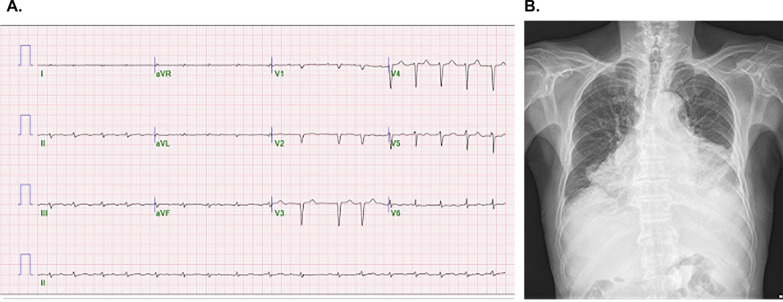
**A** Follow-up electrocardiogram shows atrial fibrillation (AF) with a heart rate of 107 bpm. **B** Follow-up chest X-ray reveals pleural effusion bilaterally

At the 1-year follow-up visit, AF was detected, with a heart rate of 107 bpm (Fig. 5A). A beta-blocker (carvedilol 3.125 mg bid) was added to the regimen for heart rate control. However, the patient visited the Cardiology Clinic within a month owing to worsening dyspnea and peripheral edema. Follow-up chest radiography revealed aggravated blunting of the bilateral costophrenic angle, indicating increased volumes of pleural effusion bilaterally (Fig. 5B). Carvedilol was discontinued immediately, and the diuretic dosage was increased (Furosemide 40 mg QD and spironolactone 25 mg QD).

At the next visit, follow-up electrocardiography revealed persistent AF, and a direct oral anticoagulant (DOAC), edoxaban 30 mg QD, was added to reduce the risk of thromboembolism associated with AF (CHA2DS2-VASc score, 4). However, the patient developed bloody stools within 2 weeks, and the gastrointestinal bleeding caused severe anemia, with a hemoglobin level of 6.4 g/dL (reference range, 13.5–17.5 g/dL). A digital rectal examination revealed internal hemorrhoids (Grade 2). The patient received two pints of packed red blood cell transfusion, and DOAC was discontinued

**Fig. 6:**
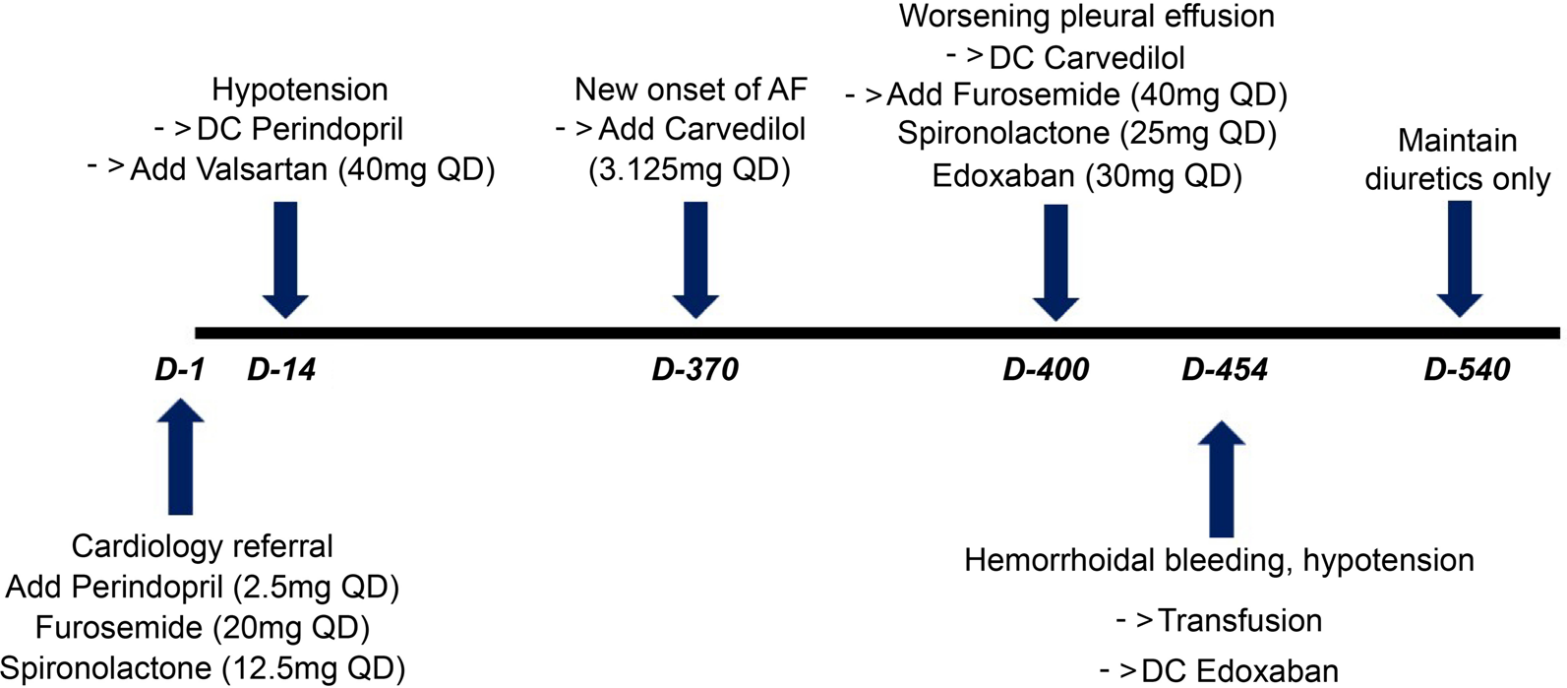
Treatment schedule of administered drugs and major complications

Follow-up TTE at one year showed deteriorated LV contractility of ejection fraction 31% and right ventricular dysfunction (RV S’ 6 cm/s). The patient was prescribed diuretics only to relieve symptoms and volume overload. The schedule of the medical treatment is summarized in Fig. 6.

## Discussion

In the field of HFpEF, ATTRwt cardiac amyloidosis has emerged as a distinct challenge, predominantly affecting the elderly population and markedly impacting their health [4]. Diagnosing cardiac amyloidosis traditionally prompts invasive procedures, such as endomyocardial biopsy, a method particularly unsuitable for older patients owing to its associated risks. The diagnosis of ATTRwt in older populations has been recently facilitated through nuclear scintigraphy for the early detection of cardiac involvement [5]. This imaging modality is a pivotal diagnostic tool that enables the precise identification of ATTR cardiac amyloidosis without intrusive interventions [6]. Its significance lies in its accuracy and accessibility, offering a safer alternative for the prompt establishment of diagnosis in older patients.

Despite recent diagnostic advancements, managing older patients with ATTRwt remains challenging. The age-dependent penetrance of ATTRwt cardiac amyloidosis introduces intricacies in its diagnosis and management, prompting a nuanced approach toward the unique needs of older individuals. Addressing these complexities requires a comprehensive strategy that encompasses enhanced accessibility to diagnostic methods, increased awareness among healthcare providers, and ongoing studies aimed at refining diagnostic techniques and therapies. This case of an 83-year-old man underscores the challenges in managing symptomatic cardiomyopathy attributed to ATTRwt cardiac amyloidosis. Frequent hypotension, new onset of AF, intolerance to beta-adrenergic blockers, and bleeding episodes emphasize adequate medication management in affected individuals. In another case report by Skov et al. [7], an elderly patient with ATTRwt presented with frequently encountered cardiac comorbidities, including AF, ventricular arrhythmia, and ischemic heart disease. This aligns with our case, where the patient showed symptoms of heart failure and later developed AF, underscoring the arrhythmogenic potential of ATTRwt.

Patients with cardiac amyloidosis have a high prevalence of AF of up to 88% [6]. In addition, these patients have a high incidence of thromboembolism independent of the CHA2DS2-VASc score and left atrial volume index [8]. The management of AF in our patient with ATTRwt presented the clinical team with complex decision-making, particularly for the choice of medication because low-dose beta-adrenergic antagonists and oral anticoagulant therapies exacerbated the condition.

Beta-adrenergic blockers are well-known non-tolerated drugs in patients with cardiac amyloidosis. In contrast, a Spanish prospective registry study showed that beta-adrenergic antagonists are associated with lower mortality rates in patients with cardiac amyloidosis [9]. However, their use in cardiac amyloidosis is limited to patients with early-stage diastolic dysfunction, and the majority develop drug intolerance [10, 11]. Because of the restrictive physiology of cardiac amyloidosis, the stroke volume is fixed; therefore, cardiac output depends on the heart rate. Beta-adrenergic antagonists reduce heart rate and cardiac output, resulting in drug intolerance [12]. Similarly, conduction disorder is common in cardiac amyloidosis and occurs in up to 43% of patients with ATTRwt [13]. Therefore, the use of beta-adrenergic blockers in cardiac amyloidosis needs monitoring because they can precipitate conduction disorders and inhibit compensatory tachycardia for adequate cardiac output [14].

In our case, the patient developed intolerance to the initial therapy for AF with a low-dose beta-blocker, which manifested as dyspnea and fatigue, coupled with increased volume overload. The beta-blockers were unanimously discontinued upon recognizing the adverse effects. Subsequently, the patient’s symptoms notably improved. This emphasizes the significance of a delicate balance when prescribing beta-adrenergic antagonists to older patients with ATTRwt. Beta-adrenergic antagonists may induce hemodynamic intolerance and increase the risk of bradycardia in patients with cardiac amyloidosis. This is particularly concerning in a heart likely prone to conduction system disorders and may rely on compensatory tachycardia for adequate cardiac output [15]. The tolerance of amyloidosis to beta-adrenergic antagonists has yielded mixed findings in clinical studies, with some suggesting a potential benefit regarding all-cause mortality, whereas others have not replicated these findings [9].

Previous studies have reported that oral anticoagulants can be safely used to treat TTR amyloidosis in the absence of severe renal failure [16]. In our case, the patient developed hemorrhoidal bleeding within two weeks of initiating low-dose DOAC therapy. In amyloidosis, the risk of bleeding can increase because of amyloid involvement in the digestive and hematological systems or amyloid angiopathy [17]. However, other conditions common in older patients, such as the presence of comorbidities, fragility, frequent drug-to-drug interactions, and decreased renal function, can also induce the development of bleeding complications in patients with amyloidosis [18].

The patient exhibited tolerance to angiotensin-converting enzyme inhibitors, ARBs, MRAs, and diuretics. However, as the systolic dysfunction deteriorated, hypotension developed; therefore, low-dose ARBs were discontinued. MRA and diuretics were administered as part of the treatment regimen. Although we did not prescribe sodium-glucose cotransporter 2 inhibitor, sodium-glucose cotransporter 2 can improve patients symptom and volume status. The patient's responses to these therapies highlighted the individual variability in responses to the drugs and the need for personalized treatment strategies for ATTRwt in the older population.

## Conclusion

This case emphasizes the need for a personalized approach in managing ATTRwt in older populations, where the choice of medication should be carefully considered owing to the patient’s unique presentation and intolerance. Further studies and collaborative efforts are essential to refine the treatment strategies and improve the outcomes in this patient population.

## Data Availability

Not applicable as no new data were generated.

## Abbreviations

ACEI: Angiotensin-converting enzyme inhibitor
AF: Atrial fibrillation
ARB: Angiotensin receptor blocker
ATTRwt: Wild-type transthyretin amyloidosis
CMR: Cardiac magnetic resonance
DOAC: Direct oral anticoagulant
HFpEF: Heart failure and preserved ejection fraction
LV: Left ventricle
MRA: Mineralocorticoid receptor antagonist
TTE: Transthoracic echocardiography
TTR: Transthyretin
99mTc-PYP: Technetium pyrophosphate
